# Diagnosis of Behçet’s disease: clinical characteristics, diagnostic criteria, and differential diagnoses

**DOI:** 10.1186/s41927-020-00172-1

**Published:** 2021-01-15

**Authors:** Mina Kiafar, Seyedeh Tahereh Faezi, Amir Kasaeian, Azarakhsh Baghdadi, Sahar Kakaei, Seyed Ali Mousavi, Mohammad Nejadhosseinian, Farhad Shahram, Seyedeh Zahra Ghodsi, Hormoz Shams, Fereydoun Davatchi

**Affiliations:** 1 Rheumatology Research Center, Tehran University of Medical Sciences, North Amirabad Street, Tehran, 1411713137 Iran; 2 Valiasr Education and Treatment Center, Zanjan University of Medical Science, Zanjan, Iran; 3 Hematology-Oncology and Stem Cell Transplantation Research Center, Tehran University of Medical Sciences, Tehran, Iran; 4 Internal Medicine Center, Zahedan University of Medical Sciences, Zahedan, Iran; 5 Department of Dermatology, Razi Hospital, Tehran University of Medical Sciences, Tehran, Iran

**Keywords:** Behçet disease, Clinical manifestations, Disease manifestation, Diagnosis, Iran

## Abstract

**Background:**

The diagnosis of Behçet disease (BD) is challenging in many cases. The purpose of this study was to describe the clinical characteristics of patients at a referral BD clinic.

**Methods:**

In a retrospective study, we collected data from patients at a national referral Behçet clinic from November 2018–August 2019. A BD diagnosis was confirmed (BD group) or ruled out (Non-BD group), and the two groups were compared for differences.

**Results:**

A total of 238 patients satisfied the inclusion criteria. Forty patients (16.8%) were finally diagnosed with BD. Ocular and genital lesions were significantly more prevalent in the BD group. A positive pathergy test and HLA-B51 were also significantly more common in BD. However, oral lesions, articular involvement, and gastrointestinal manifestations were similar between groups. Also, patients with BD were significantly more likely to have multi-organ (≥2 organ systems) involvement.

**Conclusions:**

Being the first study to evaluate the clinical characteristics of patients who are visited at a referral BD clinic and are believed to have a high probability of Behçet, the results of this study are important from an epidemiological standpoint. Also, the findings of this study could be used by referral Behçet clinics, which evaluate and diagnose patients with a high pretest probability and atypical presentations of BD on a daily basis. The alternative diagnoses established in this study could be used as the list of the most common differential diagnoses for Behçet’s disease.

## Background

Behçet disease (BD) is an inflammatory vasculopathy with multisystemic involvement. The clinical course usually follows a relapsing-remitting course with heterogeneous clinical manifestations [1]. Despite extensive research dedicated to the underlying mechanisms of BD, we still have a long way to understand the complexity of Behçet disease. While the etiology is unknown, a strong correlation with human leukocyte antigens, specifically HLA-B51, has been observed. Behçet disease has been reported all over the world, but the prevalence is particularly high in the middle east, far east, and the Mediterranean. BD is also referred to as the ‘silk route disease’, acknowledging the fact that the highest incidence of BD has been reported along this ancient route. Turkey has the highest prevalence of BD, followed by Iran, Saudi Arabia, Iraq, Israel, northern China, and Korea [2, 3].

There are no pathognomonic laboratory tests to diagnose BD, and as such, the diagnosis is based on clinical criteria. Clinical manifestations of BD are heterogeneous and may involve virtually all organ systems. BD was first described as a dermatologic disease, and the mucocutaneous lesions are the hallmarks of the disease. However, ocular, cardiovascular, articular, neurological, and gastrointestinal manifestations are also common and may be present simultaneously or not, which adds to the difficulty of reaching the diagnosis [3–5]. The International Criteria for Behçet Disease (ICBD) was born from the collaboration of experts from 27 countries to address the diagnostic dilemma and the shortcomings of the previous criteria [6]. One lesson from this collaborative effort is that while the ICBD criteria is highly sensitive and specific, there are still differences in clinical manifestations of the disease among ethnicities and countries.

Patients at Behçet referral centers might have a different and occasionally have atypical presentation, which renders the diagnostic approach more complicated. There is a knowledge gap in how the current diagnostic criteria perform in the setting of a dedicated ambulatory BD center. We, therefore, designed this study to characterize the clinical manifestations of the patients referred to a national, multidisciplinary referral Behçet clinic and to determine the discriminatory characteristics in patients with Behçet disease.

## Methods

In a retrospective cohort study, data from patients referred to the Behçet clinic of the Rheumatology Research Center (RRC) at Tehran University of Medical Sciences were reviewed from November 2018 to August 2019. RRC is the only national referral center for BD and is the national authority on guidelines for prevention, diagnosis, and treatment of Behçet disease. A remarkably high percentage of patients suspected for the diagnosis are referred to RRC from all over the country, and as such, would provide a nationally representative sample of patients suspected for, and diagnosed with BD. Inclusion criteria included first-time referral from doctors of medicine, and complete follow-up records, at least until the diagnosis was proved or ruled out. Patients with a known diagnosis of BD, and those with incomplete records or follow-ups, were excluded.

The specialty of the referring physician, as well as their practice setting (academic, non-academic), were collected. Demographic data, including age, gender, and ethnicity, were recorded. A complete history of the current symptoms was obtained, which included type, severity, and duration of symptoms, as well as the family history of BD and drug history. Subsequently, all patients were examined by a team of multidisciplinary BD experts, including rheumatologists, dermatologists, and ophthalmologists. A thorough and systematic examination looking for mucocutaneous lesions, and ocular, articular, cardiovascular, and neurological involvement was performed. Articular symptoms were further evaluated with a focused physical examination and radiographs where required, to distinguish between various etiologies. A Pathergy test was done for all patients. Results of laboratory tests, including HLA-B5, HLA-B51, and HLA-B27, were recorded.

The ICBD criteria were used to diagnose/rule-out BD, dividing patients into two groups (BD, Non-BD), which were subsequently compared. The definitive diagnosis of patients who did not meet the ICBD criteria was also reviewed. For patients with a BD diagnosis, organ systems involved, disease severity, and the subspecialty consult requested at the first visit were recorded.

Descriptive statistics were used to determine the frequencies and central tendencies of the cohort. Groups were compared utilizing the Student *t*-test and Mann–Whitney U test for parametric and non-parametric data. Chi-Square test was used to compare categorical data. All statistical analyses were performed with IBM SPSS Statistics for Windows, Version 22.0 (IBM Corp., Armonk, NY). A *P* value of < 0.05 was considered significant.

The study protocol was approved by the Ethics Committee of Tehran University of Medical Sciences (IRB 1398.397, 8/13/2019). All subjects were informed about the study goal and methods. They were enrolled after their written informed consent.

## Results

During the one-year period of this study, a total of 238 patients met the inclusion criteria. One-hundred thirty-one (55%) were female, and 107 (45%) were male. The mean age of the referred patients was 37.4 years (range, 10–74, SD 12.8). The most common ethnicity among patients was Turk (93 patients, 39%), followed by Fars (92 patient, 38.7%), Kurd (23 patients, 9.7%), Lor (22 patients, 9.2%), Afghan (6 patients, 2.5%), and Turkman, Balouch, and Arab each with one patient (0.4%).

The most common symptom for which patients were referred, was oral lesions (in 204 patients, 85%), which was followed by ocular lesions (20 patients, 8.4%), genital lesions (11 patients, 4.6%), back pain (4 patients, 1.7%), articular involvement (4 patients, 1.7%), and other skin lesions (4 patients, 1.7%). Patients were symptomatic for a mean 91.7 months (range, 10 days to 408 months, SD 94.2 months) before their visit at our center. For patients with multiple symptoms, the duration of their earliest symptoms was used. Twenty-three patients (9.7%) had a family history of BD, and 72 patients (30.3%) had a family history of recurrent oral aphthosis.

Rheumatology was the most common specialty of the referring physicians (91 patients, 38.2%), followed by internal medicine (63 patients, 26.5%) and ophthalmology (26 patients, 10.9%). Referring physicians were most commonly practicing in a non-academic center (87 physicians, 36%).

Table 1 summarizes the frequency of symptoms/signs on the physical examination, as well as the results of qualitative laboratory tests, including HLAs. Single-organ involvement was present in 114 patients (48%), followed by two organ systems (51 patients, 21.4%), and three organ systems (20 patients, 8.4%).

**Table 1:** Results of the physical examinations and laboratory tests at the initial evaluation

Symptom/Sign/Lab test	No. positive (Percent)	Comments
Oral lesions	219 (92%)	Most common: ≤3 round lip lesions, 1-5 mm in size
Ocular lesions	105 (44.1%)	Posterior uveitis was the most common pathology (49 patients), followed by anterior uveitis (25 patients)
Genital lesions	87 (36.6%)	
Articular involvement	38 (16%)	Mechanical joint pain was more common than inflammatory arthritis
Gastrointestinal	31 (13%)	Diarrhea was the most common symptom (15 patients), followed by nausea, and rectorrhagia
Neurologic involvement	17 (7.3%)	Seizure was the most common (9 patients)
Erythema Nodosum	15 (6.3%)	
Cardiovascular	13 (5.6%)	DVT in 5 patients and pulmonary thromboembolism in 3 patients
Pseudofolliculitis	4 (1.7%)	
Positive Pathergy reaction	30 (12.6%)	A positive pathergy reaction was the reason for referral in 5 patients
Human Leukocyte antigens		
HLA-B5	138 (58%)	
HLA-B51	114 (47.9%)	A positive HLA-B51 antigen was the primary reason for referral in 35 patients
HLA-B27	15 (6.3%)	

Note that total is > 100% due to multi-organ involvement

*DVT* Deep-vein thrombosis, *HLA* Human leukocyte antigen

### Diagnosis of BD

The ICBD criteria were used to determine a diagnosis of BD (Table 2). Patients with a score of < 3 were considered as not having BD. A score of 3 was considered probable BD, and a score of ≥4 indicated a definitive diagnosis of BD. Forty patients (16.8%) were diagnosed with BD, 105 patients (44.1%) had a probable diagnosis, and BD was ruled out in 93 (39.1%) patients.

**Table 2:** The international criteria for Behçet disease scoring system

Sign/Symptom	Points
Ocular lesions	2
Genital aphthosis	2
Oral aphthosis	2
Skin lesions	1
Neurological manifestations	1
Vascular manifestations	1
Positive pathergy test^a^	1

^a^ Pathergy test is optional. Where a pathergy test is conducted, 1 extra point may be added for a positive result. Adapted from International Team for the Revision of the International Criteria for Behçet’s Disease (ITR-ICBD). The International Criteria for Behçet’s Disease (ICBD): a collaborative study of 27 countries on the sensitivity and specificity of the new criteria. Journal of the European Academy of Dermatology and Venereology. 2014 Mar;28(3):338–47 [6]

The final diagnoses of patients in whom BD was ruled out are listed in Table 3. It should be noted that in 26 patients in the non-BD group, we did not establish a specific diagnosis. As these patients were referred for a second opinion regarding BD, they were not followed to see whether a subsequent diagnosis was made by the referring physician.

**Table 3:** The final diagnosis in patients in whom a diagnosis of Behçet disease was ruled out

Diagnosis	No. patients (percent)
Simple aphthous lesions	45 (18.9%)
Lichen Planus	12 (5%)
Isolated ocular involvement	5 (2.1%)
Pemphigus	3 (1.3%)
Geographic tongue	2 (0.8%)
Herpes simplex	2 (0.8%)
No alternative diagnosis was made	26 (10.9%)

### Post-diagnosis analysis

After applying the ICBD criteria, patients were divided into two groups for further comparison: those with a definite diagnosis of Behçet disease (BD, 40 patients), and those in whom BD was ruled out (Non-BD, 93 patients). There was no significant difference between the two groups in terms of age, gender, and ethnicity (all *P* values > 0.05). The duration of symptoms was also similar between groups (*P* = 0.58). In both groups, oral lesions were the most common reason for referral, and the presenting symptoms were not significantly different between groups (*P* = 0.79).

Patients in the BD group had a significantly higher prevalence of a family history of BD, 9/40 compared to 5/93 (*P* < 0.001). They also had a significantly higher prevalence of a family history of oral aphthosis, 23/40 compared to 20/93 (*P* < 0.001).

When comparing the specialty of the referring physician, ophthalmologists were significantly more likely to refer a patient with the final diagnosis of BD (*P* < 0.001). The academic and non-academic practice was not a significant predictor (*P* > 0.05). The notable history and physical examinations of the BD and Non-BD groups, as well as the results of pertinent lab tests, are summarized in Table 4. Refer to Table 1 for comparison to the frequencies within the entire study population.

**Table 4:** Comparison between the BD and Non-BD groups

Symptom/Sign/Lab test	BD group, No. positive (percent)	Non-BD group, No. positive (percent)	*P*-value
**Oral lesions**	**40 (100%)**	**83 (89.25%)**	**0.031**
**Genital lesions**	***26 (65%)***	***17 (18.28%)***	***< 0.001***
**Ocular lesions**	***20 (50%)***	***13 (13.98%)***	***< 0.001***
**Erythema Nodosum**	**11 (27.5%)**	**0 (0%)**	**< 0.001**
Articular involvement	10 (25%)	13 (14%)	0.123
**Pseudofolliculitis**	**3 (7.5%)**	**0 (0%)**	**0.008**
Gastrointestinal	3 (7.5%)	12 (12.9%)	0.18
Cardiovascular	2 (5%)	2 (2.1%)	0.51
Neurologic involvement	1 (2.5%)	2 (2.1%)	0.9
***Positive Pathergy reaction***	***18 (45%)***	***0***	***< 0.001***
Leukocyte antigens			
HLA-B5	26 (65%)	46 (49%)	0.09
***HLA-B51***	***24 (60%)***	***36 (38%)***	***0.02***
HLA-B27	3 (7.5%)	9 (9.6%)	0.68

History, physical examination, and laboratory tests compared between BD and Non-BD groups, sorted by decreasing frequency in the BD group. Significant comparisons are indicated in bold

A positive pathergy test (PPT) is an optional score in the ICBD criteria, and therefore, might not be performed by all clinicians. As such, and considering the fact that we routinely perform a pathergy test for our patients, we sought to determine how many patients benefited from the addition of a PPT. Only two patients had a score of 3 prior to the pathergy test, both of whom had oral and skin lesions only. In these cases, a PPT added the 1 score needed to establish a BD diagnosis. In the other 38 patients (95%), a diagnosis of BD was made regardless of the result of the pathergy test.

We also evaluated the performance of the legacy diagnostic criteria of the International Study Group for Behçet’s disease (ISG) [7]. The ISG criteria defined Behçet’s disease as recurrent oral ulceration plus two of the following: recurrent genital ulcerations, eye lesions, skin lesions, or a positive pathergy test. When considering the ICBD criteria as the gold-standard, ISG was only able to detect 26/40 (65%) BD cases. False negative results all had two criteria, with 10/14 (71%) having oral and ocular lesions, and 4/14 (29%) having oral and genital ulcers, neither of which is defined as BD with the ISG criteria.

As seen in Table 4, oral, ocular, and genital lesions, as well as Erythema nodosum and Pseudofolliculitis were significant predictors for the presence of BD. The details of ocular involvement are listed in Table 5.

**Table 5:** Details of ocular involvement in patients with and without a Behçet disease diagnosis

Ocular lesion	BD group, No. positive (percent)	Non-BD group, No. positive (percent)	*P*-value
***Anterior uveitis***	***8 (20%)***	***6 (6.4%)***	***0.02***
***Posterior uveitis***	***24 (60%)***	***8 (8.6%)***	***< 0.001***
***Panuveitis***	***8 (20%)***	***3 (3.2%)***	***0.001***
***Retinal vasculitis***	***6 (15%)***	***2 (2.1%)***	***0.004***
Panophthalmitis	1 (2.5%)	0	0.12
Other lesions	1 (2.5%)	1 (1%)	0.53

Significant comparisons are indicated in bold

Multi-system involvement was significantly more common in BD patients compared to the Non-BD group (*P*< 0.001). While 80% of the patients in the BD group had ≥2 organ systems involvement, compared to only 8.6% of the patients in the Non-BD group.

## Discussion

Behçet disease has been a diagnostic dilemma since the first descriptions of the disease were published. The evolution of the diagnostic criteria in the last two decades, which has only been possible with a massive international collaboration, has led to the birth of ICBD, which is the most sensitive and specific diagnostic criteria yet [8]. In this study, we approached this dilemma from a different perspective. We tried to answer the following question: when faced with a population of patients with a high probability of BD, which features (clinical exam/lab tests) are most predictive of a Behçet disease diagnosis? To the best of our knowledge, this is the first study with this novel approach, which was only made possible by being the single nationally-renowned referral Behçet clinic in the country.

As expected, oral lesions were the most common reason for referral, as well as the most prevalent manifestation in patients finally diagnosed with BD. Oral aphthosis is the most sensitive symptom of BD, and has been reported in 90–100% of patients in national reports [3, 9–12]. However, due to the high prevalence of recurrent aphthous stomatitis in the general population, which has been reported as high as 20%, oral lesions are neither specific nor pathognomonic for BD [1, 13, 14]. As evident in Table 4, oral lesions might not be discriminatory in cases with a high pretest probability, which in this study, were the patients referred with a high suspicion for BD by medical specialists. Letsinger et al. reported a cohort of 64 patients with complex aphthosis who were referred to be evaluated for BD [14]. Only 10 patients were diagnosed with BD in that study. Our results also show a similar pattern, with a high prevalence of oral aphthosis in patients with and without BD. Additionally, simple aphthosis was the most common final diagnosis in patients in whom BD was ruled out. Lichen planus, isolated ocular lesions, pemphigus, geographic tongue, and herpes simplex were the other diagnoses in this cohort, which could be an accurate estimate of the most common differential diagnoses of BD.

In contrast, genital and ocular lesions were significant predictors of BD in high-probability patients. Both genital and ocular manifestations have a heterogeneous prevalence among studies, with large ethnic and regional differences [3, 15]. However, they are more specific than oral lesions. Uveitis is the most common ocular manifestation in BD [2, 15, 16], which was also the case in our study. We found that not only ocular lesions in BD are highly specific in patients evaluated for BD, but also patients referred by ophthalmologists are significantly more likely to be diagnosed with BD. The same was not true for other specialties, even for rheumatology. It should also be noted that the pattern of the referring physicians in this study might not be generalizable to what is practiced worldwide. A significant percentage of our fellowship graduates have a private practice and are the main referral source for our BD clinic. In contrast, the most common pattern in other countries is that BD patients are referred by dermatologists practicing in the academic setting.

A positive pathergy test is an optional criterion in the ICBD, for two main reasons: the test is not part of the routine care of patients in many countries. Also, there is no standardized protocol to administer the test, which decreases its predictive power. That being said, a positive pathergy test was the most specific diagnostic tool in this study. While only 45% of the BD patients had a positive result, none of the Non-BD group showed a positive reaction. A pathergy test is a part of our routine evaluation of patients for Behçet disease. We suggest unifying the protocol for the administration of the pathergy test, which has been shown to be a highly predictive test.

HLA-B51 is more prevalent in BD than the general population [17]. Due to low specificity and a variable prevalence among ethnicities, it is not considered a diagnostic criterion in the ICBD [6]. However, among BD patients in this study, a significantly higher percentage were positive for HLA-B51 than patients in whom BD was ruled out. This was not the case for HLA-B5 and HLA-B27. Sixty percent of BD patients in this study were positive for HLA-B51, while previous studies have determined a 27% prevalence of an HLA-B51 genotype in the general population in Iran [3].

This study has some limitations. First, not all patients suspected of BD are referred to our institution. Patients with a milder disease might have been managed without a referral, and we might thus be biased towards more severe patients with non-typical presentations. Also, patients from a lower socioeconomic status might have difficulties in access to health care. Additionally, this was a retrospective cohort study, and therefore, the follow-up of patients in terms of treatment and prognosis was not done. Therefore, as the manifestations of BD might be accumulative in time, there is still a possibility that some patients would later be diagnosed with BD. This study also has some strengths. This is the first study of a large national Behçet center to look for the characteristic of patients referred for evaluation and not only those with a definite BD diagnosis. Also, all patients were examined by a multidisciplinary team, and the risk of misdiagnosis was very low.

## Conclusions

To conclude, in a study of 238 patients referred to a national Behçet clinic, 16.8% of patients were finally diagnosed with Behçet’s disease. In this cohort of patients, who presumably had a high probability of Behçet’s disease, ocular and genital lesions, together with a positive pathergy test and HLA-B51, are significantly more prevalent in those who are finally diagnosed with Behçet’s disease. Also, patients were significantly more likely to have multi-organ (≥2 organ systems) involvement. The results of this study could be used by referral Behçet clinics that face patients with a high probability of Behçet’s disease and atypical presentations on a daily basis. Also, the alternative diagnoses established in this study could be used as the list of the most common differential diagnoses for Behçet’s disease.

## Data Availability

The datasets used and/or analyzed during the current study are available from the corresponding author on reasonable request.

## Abbreviations

BD: Behçet’s disease
Non-BD: Non-Behçet disease. Patients in whom the diagnosis of BD was ruled out
HLA: Human Leukocyte Antigen
ICBD: The International Criteria for Behçet Disease
RRC: Rheumatology Research Center
DVT: Deep vein thrombosis
